# Habits of Mind as a Catalyst for 21st Century Skills and Environmental Sustainability: A Bibliometric Analysis with a Focus on SDG 6 in Educational Research

**DOI:** 10.12688/f1000research.170682.2

**Published:** 2026-01-13

**Authors:** Ella Izzatin Nada, Sajidan Sajidan, Sri Retno Dwi Ariani

**Affiliations:** 1 Department of Natural Science Education, Universitas Sebelas Maret, Surakarta, Indonesia; 2 Department of Chemistry Education, Faculty of Science and Technology, UIN Walisongo, Semarang, Indonesia; 3 Department of Biology Education, Universitas Sebelas Maret, Surakarta, Indonesia; 4 Department of Chemistry Education, Universitas Sebelas Maret, Surakarta, Indonesia

**Keywords:** Habits of Mind, 21st Century Skills, Environmental Sustainability, SDG 6, Bibliometric Analysis, Education.

## Abstract

Habits of mind have increasingly been recognized as foundational dispositions for developing 21st-century skills and supporting sustainability-oriented education. This study presents a systematic literature review combined with bibliometric analysis to map the intellectual structure, research trends, and thematic evolution of habits of mind in educational research, with particular attention to sustainability and Sustainable Development Goal 6 (SDG 6: Clean Water and Sanitation). A Scopus-based dataset of peer-reviewed publications published between 2010 and 2025 was analyzed using co-authorship, co-citation, and keyword co-occurrence techniques implemented in VOSviewer. The analysis identifies dominant research clusters related to critical thinking, STEM education, teacher education, and sustainability-oriented learning, as well as leading authors, journals, and countries contributing to the field. While sustainability-related themes are increasingly visible, explicit connections between habits of mind and SDG 6 related educational issues remain limited and unevenly distributed, particularly in the Indonesian context. The findings highlight underexplored research areas and reveal opportunities for strengthening the integration of cognitive dispositions, sustainability education, and water sanitation awareness through culturally responsive educational strategies. This study contributes by providing a structured overview of the field and clarifying research gaps to guide future educational research and policy aligned with SDG 6.

## Introduction

Education plays a central role in human capital development and in equipping learners with the cognitive dispositions required to navigate complex social, economic, and environmental challenges ( Erjavec, 2020; Olaniyan & Okemakinde, 2008). In recent decades, increasing attention has been given to habits of mind such as critical thinking, persistence, creativity, and self-regulation as foundational dispositions underlying 21st-century skills. These dispositions support learners’ ability to adapt, collaborate, and engage in problem solving within rapidly changing and uncertain contexts.

Beyond employability and innovation, contemporary education is increasingly expected to contribute to sustainability agendas. Education for Sustainable Development emphasizes systems thinking, ethical reasoning, and reflective judgment as essential capacities for addressing environmental challenges ( Lander, 2015). Among the Sustainable Development Goals, SDG 6 (Clean Water and Sanitation) represents a critical global priority, particularly in countries facing persistent water access and sanitation challenges. In Indonesia, disparities in water quality and sanitation infrastructure remain pronounced, especially in rural and remote regions ( Wulandari et al., 2024), underscoring the need for educational approaches that foster environmental awareness and responsible resource use.

Although habits of mind, 21st-century skills, and sustainability education have each been examined in prior research, existing studies remain fragmented. Previous empirical studies and reviews tend to focus on cognitive dispositions in isolation (e.g., critical thinking in STEM education or teacher education) without systematically examining their intersection with sustainability-oriented educational goals. Moreover, existing bibliometric and systematic reviews rarely address how habits of mind are positioned within sustainability education or linked to specific SDGs, nor do they adequately represent evidence from emerging economies such as Indonesia. This gap limits the understanding of how cognitive dispositions are mobilized in educational research to support sustainability agendas.

To address this gap, the present study conducts a systematic literature review combined with bibliometric analysis to map the global research landscape on habits of mind in education, with particular attention to sustainability-related themes and SDG 6. The study is guided by the following research questions:

(1) What are the main publication trends, influential authors, journals, and countries in habits-of-mind research within education?
(2) What thematic clusters characterize the intersection of habits of mind, sustainability education, and environmental issues?
(3) How is SDG 6 reflected within this literature, and what is the position of Indonesian research within the global landscape?

By addressing these questions, this study contributes a structured overview of the field, clarifies underexplored areas, and provides a foundation for future research and educational policy development aligned with sustainability goals.

The remainder of this article is organized as follows: the next section presents the theoretical framework, followed by the methodology, results, discussion, and conclusions.

## Literature review

### Concept of habit of mind

It refers to ways of thinking and acting that individuals use as they deal with difficult problems ( Ariyati & Fitriyah, 2024; Hidayati & Idris, 2020; Susilowati, Hartini, Mayasari, et al., 2018). They are not only skills you learn but also common ways of thinking that guide us in resolving a variety of problems. If a person is said to have a good habit of mind, they are ready to work on a challenge, use critical thinking and keep going when the task challenges them. Through this approach, people get to think about how they think and use the experience to improve their solutions to problems.

The growth of these mindsets greatly benefits learning, most of all when situations are unpredictable and challenging. Cognitive dispositions which contain values, attitudes and behaviors, are important for managing hard learning tasks ( Carroll, 2012; Crick, 2009). Because today’s challenges change often and can be complex, people need to be able to think critically, be creative and stay motivated and adaptive. Learning these habits allows someone to excel in the short term and also helps them become better at learning as time passes.

Currently, habit of mind is identified as important for teaching students to adapt, a trait widely needed in today’s rapidly changing world. It was found through research in Indonesia that students planning to become teachers mostly rely on self-regulation, while critical thinking and creative thinking are the next most important habits ( Ariyati & Fitriyah, 2024). With industries and societies growing and changing, individuals have to keep up by adapting, innovating and using the skills needed to read, understand and apply new data from their environment. Being flexible with our thinking is very important now, given the rapid progress of technology. Developing this skill allows students to solve challenges well and become more creative ( Choi, 2024). For this reason, habits of mind are vital for everyone and help people handle the changing demands of their lives, jobs and active participation in society. As a result, these habits are essential for shaping people able to keep up and do well in the modern, globalized world.

### Habit of mind and its role in 21st century skill development

In recent years, there has been a growing emphasis within educational frameworks worldwide on developing 21st-century skills, which include critical thinking, creativity, collaboration, and communication. These skills are vital for success in a globalized world where innovation and complex problem-solving are essential for navigating the challenges of the future. Habits of mind are central to these frameworks because they provide the foundational cognitive and affective capacities needed to engage with and solve complex problems ( Ariyati et al., 2020, 2021). These cognitive habits foster the adaptability, resilience, and creative thinking required to innovate and tackle new challenges. Integrating habits of mind into educational curricula has been shown to improve students’ ability to apply knowledge in diverse contexts, as well as their capacity to think critically and creatively. By honing these habits, students are better equipped to approach challenges with perseverance and the ability to continuously adapt, qualities that are crucial for lifelong learning and professional success.

Several pedagogical models have been explored to nurture habits of mind in educational settings. Among the most prominent models are inquiry-based learning, problem-based learning (PBL), and experiential learning. Problem-based learning (PBL) engages students with authentic challenges, promoting interdisciplinary teamwork and the practical use of acquired knowledge ( Singha & Singha, 2024). These approaches encourage students to engage actively with real-world problems, thereby fostering reflection, critical thinking, and perseverance key components of habits of mind. Inquiry-based learning promotes curiosity and exploration, while PBL emphasizes the application of knowledge to solve practical problems, helping students develop problem-solving abilities and self-regulation skills. Experiential learning, on the other hand, provides students with hands-on, real-world experiences that reinforce cognitive and behavioral habits by encouraging direct interaction with the environment. Habits of mind such as critical thinking, creativity, and self-regulation are fundamental for sustained learning and character formation, supporting adaptive and reflective learners ( Ariyati et al., 2020; Susilowati, Hartini, Mayasari, et al., 2018).

These teaching methods not only develop intellectual capacities but also nurture emotional and social skills, such as collaboration, empathy, and self-awareness, which are equally important for personal and professional success. In particular, PBL has been widely adopted in various educational systems to cultivate habits of mind, as it requires students to work collaboratively, engage in critical thinking, and demonstrate persistence in finding solutions to complex problems. These models reflect a growing recognition that fostering habits of mind is not just about teaching content but also about developing the dispositions that allow students to thrive in a rapidly changing world.

### Habit of mind in relation to Sustainable Development Goals (SDG 6)

Researchers today are recognizing that habits of mind help promote both environmental literacy and education for sustainability, especially since the United Nations created the Sustainable Development Goal 6 (SDG 6) to emphasize clean water, sanitation and sustainable water management. Reaching the goals for sustainability such as SDG 6, greatly depends on education. During the UN Decade of Education for Sustainable Development (2005–2014), the goal was to add sustainability ideas to education across the world ( Boyle et al., 2014; Moscardo, 2015). As environmental challenges of water scarcity, pollution and climate change increase fast, it is crucial for students to learn critical thinking, how to think ethically and about systems. As a result of these habits, pupils can both comprehend and apply their understanding to problems in environmentally sound ways. If students are taught responsible water and sanitation practices, they can learn to help protect resources and the environment when they are adults.

Adding these ways of thinking to school curriculum is necessary in order for students to gain skills needed to face current and future problems in the environment ( Ardoin et al., 2020; Vira, 2019). This approach supports students to think deep about their actions and choose ways to help the environment. Thanks to systems thinking, students can see the connections among natural resources, communities and ecosystems and take a fuller view of sustainability. Since we are experiencing issues like less water or natural resources, more pollution and greater scarcity, ethical decision-making is now more essential than ever. Any education system that encourages these mindset habits will allow students to help protect the environment which helps reach SDG 6 and leads to long-term sustainability all around the world.

### Habit of mind in the context of Indonesian education

Indonesia’s educational system is currently undergoing reforms aimed at enhancing learning quality and equity across diverse regions. However, research on habits of mind remains limited, with most studies focusing on cognitive skills like critical thinking without explicitly addressing the broader dispositional habits necessary for adaptive learning. Some Research on prospective teacher students in Pontianak, Indonesia, indicates that self-regulation, critical thinking, and creative thinking are well-developed habits of mind, with self-regulation being the most dominant and creative thinking the least ( Ariyati & Fitriyah, 2024).

The Kampus Merdeka policy strives to reform Indonesia’s education system by fostering flexible, student-centered learning aligned with industry demands and individual growth, despite concerns regarding institutional readiness for implementation ( Saa, 2024; Voak et al., 2023). The challenge lies in developing culturally responsive models that integrate local wisdom with global cognitive frameworks to foster habits of mind effectively.

### Research models and approaches for developing habits of mind

Many instructional approaches can promote the growth of good habits in students in schools. Applying Inquiry Based Learning (IBL) to students motivates them to be curious, question and think deeply as they research ( Chen, 2021). Collaboration between students and critical thinking were greatly improved by the use of the Scaffolding Model in physics classes ( Susilowati et al., 2019). These approaches guide students to take charge of their thinking by planning, tracking and reflecting on what they do. Using Edutainment and Game-Based Learning, students interact with the material and grow in creativity, motivation and perseverance.

Combining these models with material that reflects Indonesian culture and life habits, supports strong habits of mind. With these methods, students can apply what they learn in class to their real lives and build skills useful in our sustainable world.

## Methodology

### Study design

This study adopts a systematic literature review (SLR) combined with bibliometric analysis, a design widely used to map research fields, identify intellectual structures, and detect emerging themes in large bodies of literature.

### Data source and search strategy

The literature search was conducted in the Scopus database, selected for its broad coverage of peer-reviewed educational and interdisciplinary research and its suitability for bibliometric analysis. The search was executed on [insert exact date] using the following query applied to TITLE-ABS-KEY fields:

(“habits of mind” OR “cognitive disposition*”) AND (“education” OR “learning”) AND (“sustainability” OR “environmental education” OR “sustainable development” OR “water” OR “sanitation”)

Publications were limited to journal articles, conference papers, and book chapters published in English between 2010 and 2025.

### SDG 6 Operationalization

SDG 6 was not treated as a strict filtering criterion at the search stage. Instead, its relevance was identified through keyword co-occurrence analysis, thematic clustering, and post-retrieval screening of sustainability- and water-related terms (e.g., water management, sanitation, clean water, environmental health) within the corpus. This approach allows SDG 6 to be examined as a thematic domain embedded within sustainability education, rather than as an isolated research category.

### Screening and selection

The initial search retrieved N records. After removing duplicates and applying inclusion and exclusion criteria, N publications were retained for analysis. A manual screening of titles and abstracts was conducted to ensure relevance to habits of mind and educational contexts. The selection process is summarized using a PRISMA flow diagram.

### Data analysis

Bibliometric analyses were performed using VOSviewer (version 1.62.0) with full counting and predefined thresholds for authors and keywords. Co-authorship, co-citation, and keyword co-occurrence analyses were conducted to identify collaboration networks and thematic clusters.

## Results

### Publication trends (2010-2025)

A steady increase in research on habits of mind in education is seen in publications between 2010 and 2025. Since around 2015, studies on cognitive dispositions have increased rapidly, as it became clear to the world that critical thinking, problem-solving and perseverance are vital to learning in the 21st century.

The highest level of income growth was seen during 2021–2023 due to how the COVID-19 pandemic affected traditional education. Because learning moved to remote and hybrid models, the significance of adaptive cognitive skills ( Lin & McNab, 2005a, 2005b) became more noticeable and researchers became more interested in studying habits of mind. They point out that there is increasing scholarly agreement that these attitudes must be part of teaching in order to help learners meet modern challenges.

**Figure 1.**
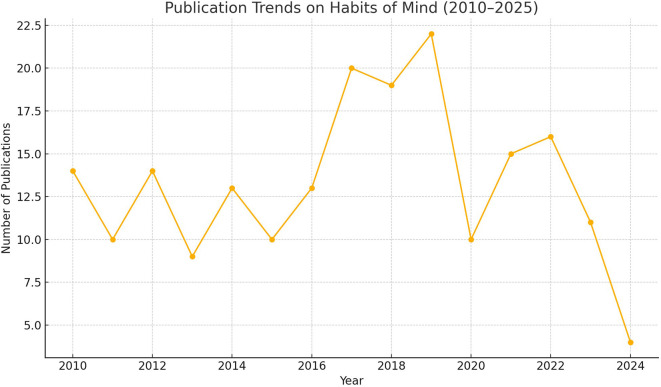
Annual publication counts on habits of mind research from 2010 to 2025.

### Highly cited papers

Using citation numbers, bibliometric analysis found the most influential publications about habits of mind in education. Published in 2015, Martin’s article is the most cited, with 582 mentions in other works, showing the great importance of making in education for encouraging both innovative and perseverant learning from students. In addition, R.W. Roeser’s (2012) research on introducing mindfulness into teacher training, cited 295 times, points out that persistence, self-control and reflection are basic skills needed in difficult learning environments.

Works like those from C. Halse (2010) on supervising doctorates, from M. Tedre (2016) concerning computational thinking and from E.R. Hollins (2011) on teacher preparation all demonstrate that cognitive abilities are necessary in different ways for students to succeed in education. They also show that habits of mind are crucial for helping both learners and educators deal with the needs of education in the 21st century.

**Table 1.** Ten most cited articles in habits of mind research.

No	Authors	Title	Year	Cites
1	L. Martin	The promise of the maker movement for education	2015	582
2	R.W. Roeser	Mindfulness Training and Teachers' Professional Development: An Emerging Area of Research and Practice	2012	295
3	C. Halse	Retheorizing doctoral supervision as professional work	2010	199
4	M. Tedre	The long quest for computational thinking	2016	181
5	E.R. Hollins	Teacher preparation for quality teaching	2011	161
6	I. Lee	Computational Thinking from a Disciplinary Perspective: Integrating Computational Thinking in K-12 STEM Education	2020	133
7	L.R. Lattuca	Supporting the Development of Engineers' Interdisciplinary Competence	2017	123
8	L. Dyche	Curiosity and medical education	2011	104
9	A. Venezia	Transitions from high school to college	2013	98
10	M. Tekkumru-Kisa	A framework for analyzing cognitive demand and content-practices integration: Task analysis guide in science	2015	97

### Leading journals and institutions

A number of vital academic journals have made research on habits of mind widely accessible by continually sharing both findings and ideas on cognitive skill growth and educational transformation. JPEER, Thinking Skills and Creativity and the Educational Psychology Review are some of the best examples. They give researchers a key opportunity to debate and advance research on how cognitive dispositions help students and adapt education to modern changes.

The regular publishing of excellent articles highlights the role of these journals in guiding the field and combining cognitive psychology, education and new innovations. Because these outlets are frequently cited in bibliometric studies of habits of mind literature, it is clear they play an important role in sharing research. Organizations in the United States have the biggest impact around the world ( Merigó & Yang, 2017).

**Figure 2.**
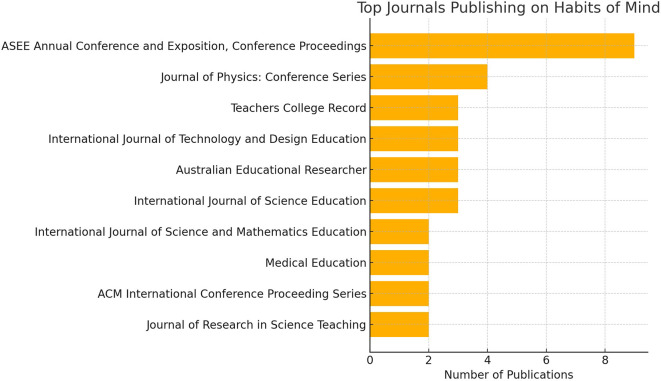
Visualization of the top ten journals publishing research on habits of mind.

### Author collaboration networks

Key contributors to the collaborative research in habits of mind are displayed clearly in the co-authorship network analysis. Researchers Lammi, M. Stephan, A.T. and Gal, I are important in the network because they help to encourage and promote joint work among peers. These networks demonstrate how institutions from many regions in North America and Europe regularly work together and exchange ideas.

Readily available research networks, including those between varying sectors and expertise, often increase the research ability and creativity of educational institutions ( Dooly et al., 2022; Ismail, 2024; Makhanya, 2020). The nature of these collaborations reveals the worldwide characteristics of habits-of-mind initiatives, reflecting how different departments and schools are contributing to knowledge together. Because these networks are strong and detailed, they show there is an active and linked academic community supporting the exchange of new ideas and the growth of unique educational methods.

**Figure 3.**
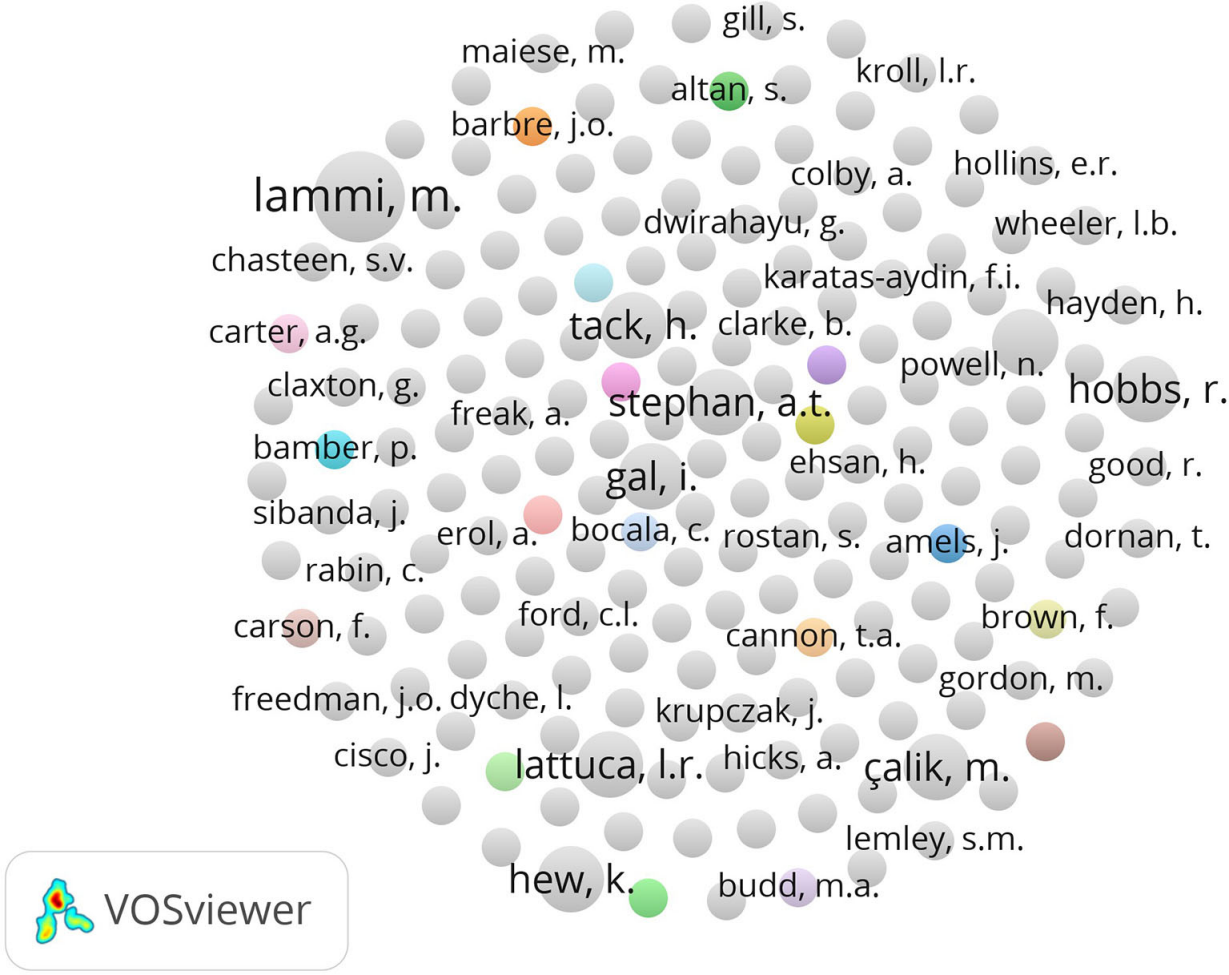
Author collaboration network in habits of mind research.

Researchers in sustainability education also form a strongly connected global community, similar to what we find in habits of mind research. Big players in sustainability research are linked in dense relationships, crossing borders and involving specialists from various fields. They show that North American and European universities mainly collaborate, sharing ideas and promoting progress in sustainability education. The strong ways people collaborate demonstrate the academic community’s joint efforts to introduce environmental education.

**Figure 4.**
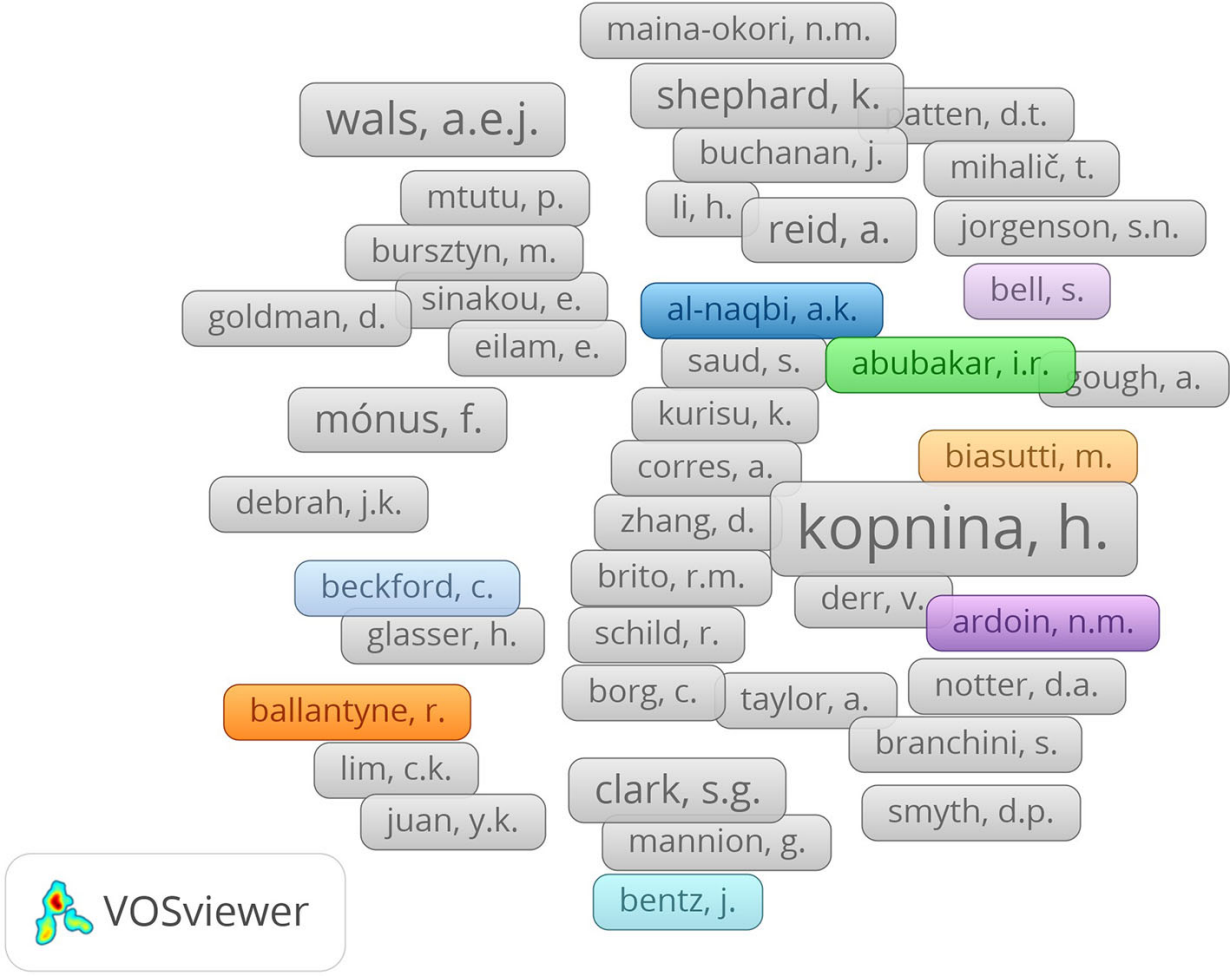
Author collaboration network in sustainability education research.

### Keyword co-occurrence and thematic emphases

In co-occurrence analysis, keywords that appear together in documents allow us to discover relationships and group together topics ( Bhuyan et al., 2021; Restrepo-Arango & Urbizagástegui-Alvarado, 2017; Sáez et al., 2023). It helps us see the main themes and central topics in the habits of mind research field. Some of the key themes in the data are mind, practice, education, critical thinking, science and development which all tend to appear with STEM education, new learning methods and professional development for teachers. The different themes underline the fact that the research covers both mental processes and learning situations.

At the same time, research connected to sustainability education shows that sustainability, environmental education and higher education are the top keywords. As a result, it’s clear that connecting thinking skills with a focus on the environment and sustainability is becoming more common. The increasing number of articles on habits of mind encourages us to believe more scholars see a connection between mental habits and solving environmental challenges in education.

**Figure 5.**
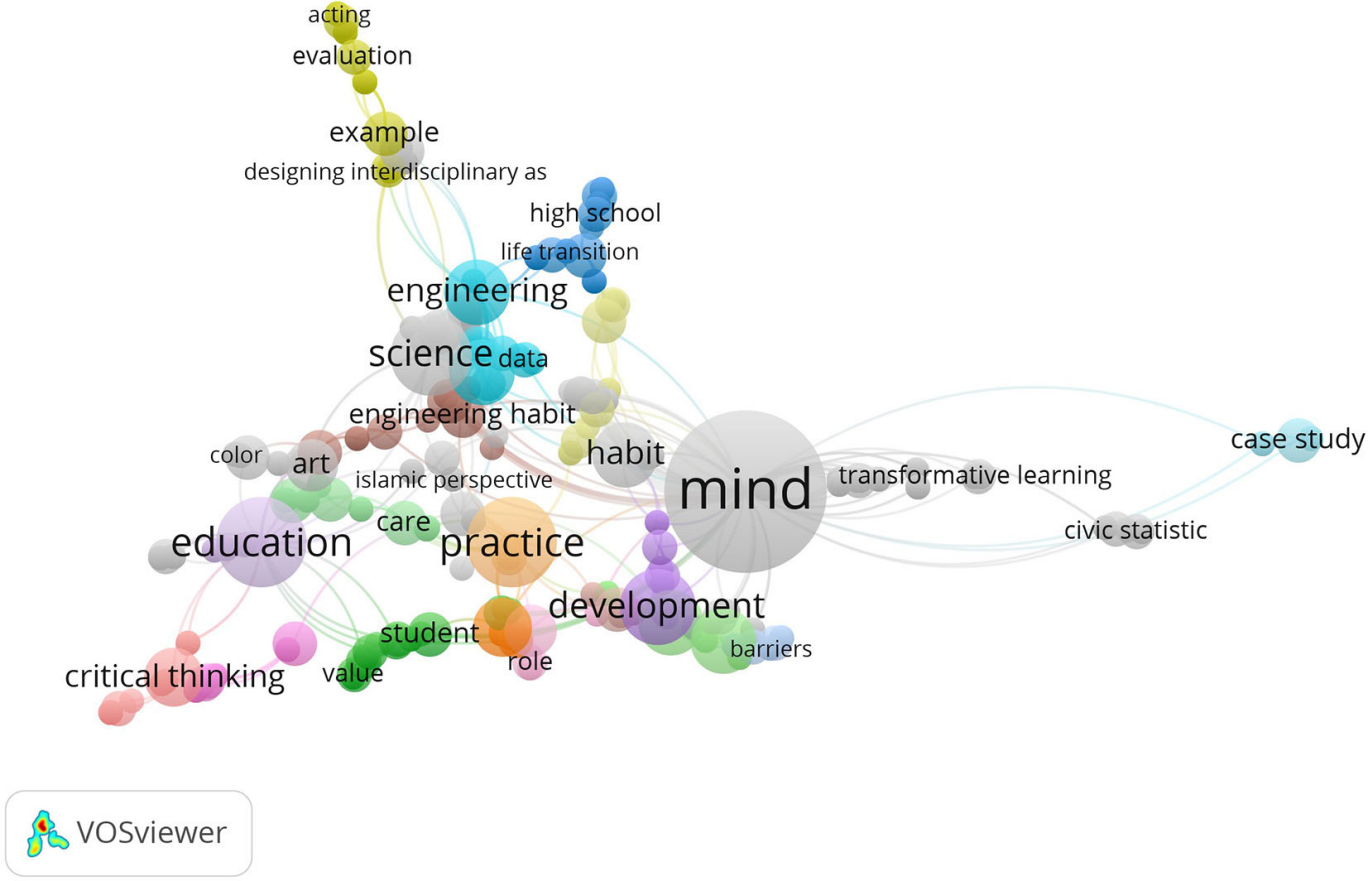
Keyword co-occurrence map in habits of mind research.

Meanwhile, when sustainability education is taken into account, researchers mostly study terms such as sustainability, environmental education and higher education. They demonstrate how we are starting to focus more on using ways of thinking that support the environment and sustainability. This means researchers are increasingly considering how habits of mind can support education aimed at solving global environmental problems. The field highlights that people’s habits make ecological crises worse or better and that using sustainable approaches is key to solving these problems ( Portera, 2022, 2023). This area’s keyword patterns show that education supports sustainable development by combining work on cognitive skills with a sense of responsibility for the environment and society.

**Figure 6.**
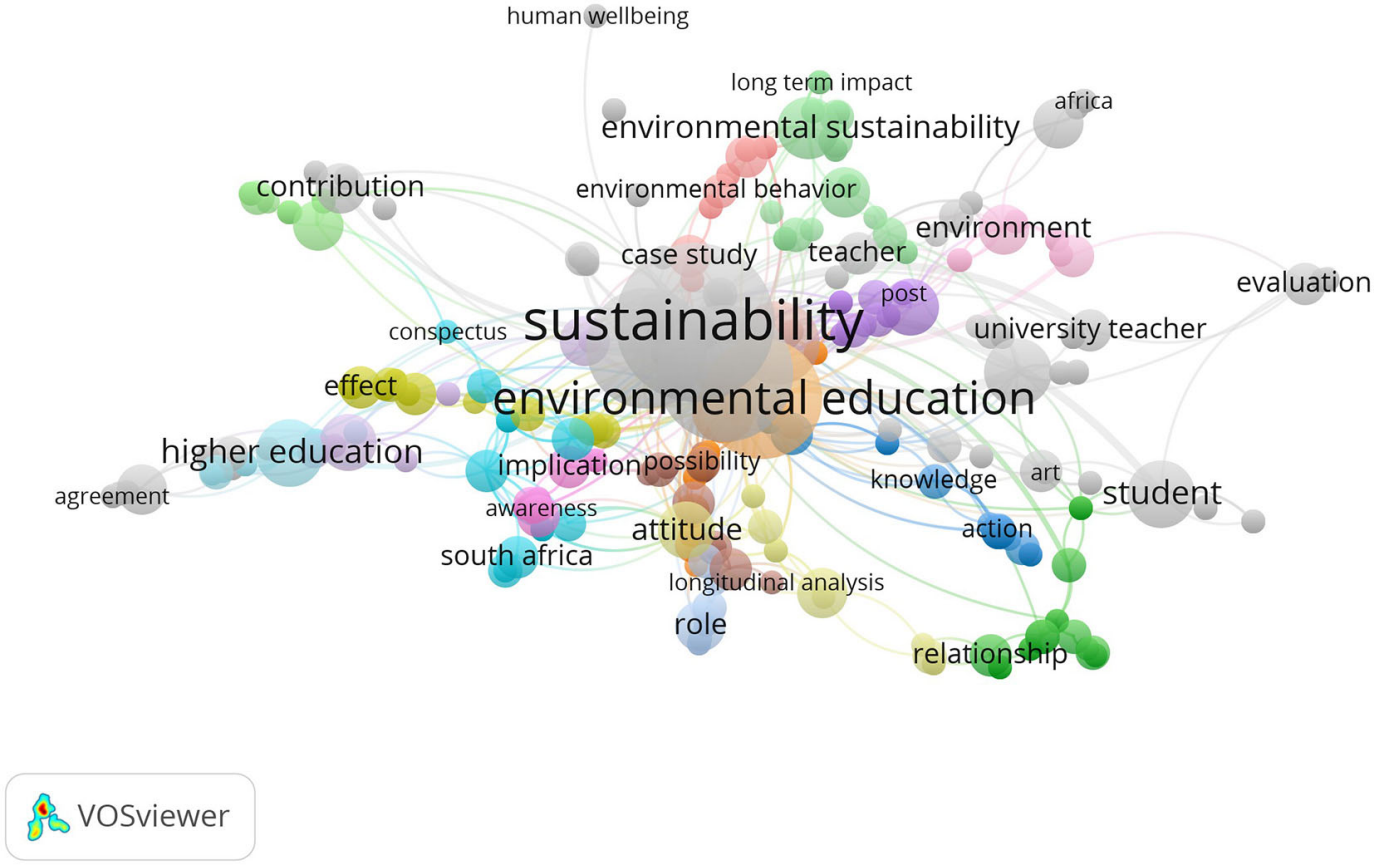
Keyword co-occurrence map in sustainability education research.

## Discussion

### Thematic analysis

Bibliometric methods have shown that several important themes guide research in habits of mind. Fostering strong thinking skills, creativity and perseverance in students is given greatest attention, as it reflects what is considered important for learners this century ( Cusanelli & Trevallion, 2020; Dwyer et al., 2014). The bibliometric cluster analysis indicates that problem-based learning and STEM-oriented pedagogies constitute one of the dominant thematic clusters in habits-of-mind research, characterized by frequent co-occurrence of keywords such as critical thinking, problem solving, and inquiry-based learning.

Moreover, improving teacher learning and revising the curriculum become essential for engraining habits of mind in schools of all kinds. This combination helps students gather information as well as put their skills into action during instruction ( Dolfing et al., 2021; Visser et al., 2010). Although progress has been made, the analysis shows that the ideas from theories are still far from being applied effectively in education at scale and in practical ways in Indonesia. Adressing this issue is important to make sure habits of mind help students learn worldwide.

### Author collaboration networks

The networks help identify important researchers and understand the structure of the academic community by showing authors who publish collaboratively and they track intellectual links and thematic clusters from how often authors are cited with each other ( Khasseh et al., 2018; Scherbakova & Bredikhin, 2021). The co-authorship network comprises multiple clusters dominated by researchers affiliated with institutions in North America and Europe, while Indonesian-affiliated authors appear in a limited number of nodes with lower network centrality, indicating emerging but still peripheral participation. The network analysis of authors shows that numerous important research clusters exist centered around key people known for their research on cognitive skills and educational psychology. These networks show a lot of collaboration among institutions within Australia, Canada, Europe, New Zealand and the United States. Both Indonesia and other emerging economies are starting to take part in global academic circles, but their roles remain fairly small.

Global teamwork and the growth of inclusive partnerships are needed to overcome existing barriers and bring about successful knowledge sharing. By collaborating more, we could speed up the development and contextual use of habits of mind research in regions often left behind which helps equalize educational innovation everywhere.

### Institutional and country collaborations

Tasks at the institutional and country levels are mainly divided between universities and research centers from North America, Europe and some parts of Asia. In North America, researchers are connected in networks that publish more, while in Europe, researchers collaborate more closely, though with less publication impact ( Danús et al., 2023). The bibliometric data indicate a growing presence of Indonesian institutions in international collaboration networks, although their overall contribution remains modest compared to institutions from North America and Europe. They greatly help encourage research methods that are sensitive to different cultures and that relate ideas from around the world to local schooling.

These efforts are necessary to fit the habits of mind ideas to the particular problems affecting Indonesian education. When international and local expertise are combined, these cooperative actions improve educational practices and support educational development in areas that are underrepresented. Adding habits of mind to what is taught at school encourages development in the mind, emotions and physical abilities ( Ariyati et al., 2020; Dwirahayu et al., 2017).

### Educational strategies

Typical educational strategies used for promoting habits of mind are inquiry based learning, PBL and experiential learning. In using these approaches, students are prompted to reflect, reason and stay motivated since these are important to growing habits of mind ( Akçay, 2009; Lower-Hoppe et al., 2020). Promoting active roles and asking for ongoing answers to problems helps learners gain the skills they need to face difficult everyday challenges.

A growing number of studies encourage embed, these instructional models in the national curricula internationally. The purpose of such integration is to boost learners’ ability to be flexible, strong and well-suited to today’s demands. As a result, many view including inquiry, problem-solving and hands-on activities within school systems as essential for growing the habits needed for continued academic success ( Narayanan, 2010; Thomas et al., 2025).

### Assessment and evaluation

To assess habits of mind, educators use tools such as self report questions, tasks that require performance and teacher reviews. To assess habits of mind, these tools look at feedback, peer assessment and self-assessment and it is peer and self-assessment that seems to connect most strongly, representing 57.1% of habit formation ( Gloria et al., 2018). These methods are used to understand both mental and emotional habits that influence each habit of mind. While progress has been made, there are many tools required that address the unique differences found in schools of developing countries.

Introduced in chemistry education to look at how students think, this approach has shown important progress in self-regulation, critical thinking and creativity ( Nahadi & Windani, 2017). It is crucial to assess existing gaps so that we can correctly measure how effective these interventions are at forming habits. As a result, more research is needed to develop, validate and adapt reliable tools for assessment, so educators and researchers have the right resources to support learning and teach evidence-based practices that are suitable for all cultures.

### Influential authors and institutions

Costa and Kallick are leading experts whose ideas have shaped most studies in the field of habits of mind. Their studies showed that cognitive dispositions are vital for good learning and problem solving. Thanks to Costa and Kallick, Habits of Mind have made a big impact on educational models meant to boost students’ abilities to think critically and solve problems. According to them, forming practices specific to learning helps children learn and grow their brains better ( Kareem et al., 2019).

Authorities in this way of thinking come from brilliant universities across the globe such as Harvard University and the University of Toronto. Universitas Sebelas Maret in Indonesia is proving to be a leading regional center which points to the increasing interest of developing nations in promoting habits of mind study. Every year, at least 10% of the revenue that is not raised by taxation is put into research and community projects by UNS and is supervised by LPPM to ensure top quality ( Kusumawardani et al., 2017). Understanding who these actors are allows us to see existing research trends and provide new opportunities for collaborations internationally.

### Emerging trends

Our study finds that there has been a major rise in research on habits of mind since 2015, with most studies focusing on critical thinking, creativity and integrating problem-based learning. Even though major countries continue to dominate publishing and citations in science, nations such as Indonesia are adding to the field each year. Although Indonesia does not dominate global publication output, the bibliometric results show a steady increase in publications from Indonesian institutions, placing Indonesia among the more active contributors within the Southeast Asian region ( Fry et al., 2023; Sukoco et al., 2023). Often, these contributions stress teaching that is fit for each culture and how effective learners are linked to helping the environment.

With the rise of these trends, there are many chances for research to use systems thinking, ethical reasoning and critical thinking to further achieve Sustainable Development Goal 6. Thinking about systems is key to resolving the closely linked problems of sustainability, especially those related to clean water and sanitation. Learning how different system components relate helps strengthen water management and sanitation ( Levy et al., 2018; Sanneh, 2018; Weaver et al., 2025). Extending research on this can result in strategies that connect mental development with saving the environment worldwide.

### Empirical findings versus conceptual implications for SDG 6

In this study, SDG 6 is treated as a conceptual and analytical lens rather than as a directly measured empirical outcome. While the bibliometric analysis identifies sustainability related themes such as environmental education, systems thinking, and responsible resource management it does not assess direct impacts on water or sanitation outcomes. Accordingly, references to SDG 6 in this discussion represent conceptual implications derived from thematic patterns in the literature, rather than causal evidence of educational interventions affecting clean water and sanitation indicators.

### Gaps in literature

Consistent with the gaps identified in the introduction, the discussion highlights a continued lack of large-scale and longitudinal studies examining how habits of mind are integrated into educational practices linked to sustainability and SDG 6, particularly in marginalized and rural contexts.

### Policy implications for Indonesia

Based on the conceptual implications derived from the bibliometric findings, Indonesian policymakers may consider introducing habits of mind into the national curriculum and teacher training programs to support the development of critical and creative thinking skills, particularly in regions experiencing the greatest educational inequality. Substantial structural challenges in remote and underdeveloped areas continue to hinder progress toward Sustainable Development Goal 6 (SDG 6). From a sustainability-education perspective, policy frameworks may therefore emphasize foundational cognitive capacities such as systems thinking, ethical reasoning, and environmental problem-solving to better equip educators to address these complex challenges ( Ariyati & Fitriyah, 2024; Nadiroh et al., 2021).

At a conceptual level, integrating SDG 6-related themes into science education may help learners develop awareness of responsible water use, resource conservation, and hygiene practices, which are commonly discussed in sustainability education literature. International research collaboration may further support the contextual adaptation of educational policies and the development of locally relevant pedagogical approaches. Aligning cognitive skill development with Indonesia’s broader economic and sustainability objectives is frequently highlighted in the literature as a pathway to ensuring that educational strategies reflect the cultural and regional diversity of the country. Prior studies have shown that cognitive skills, beyond formal schooling attainment, are associated with long-term economic outcomes such as income distribution and productivity ( Allen et al., 2018; Hanushek, 2013).

### Recommendations for future research

Building on the limitations and patterns identified in this bibliometric review, future research should prioritize the development of culturally sensitive assessment instruments to examine how habits of mind are cultivated through environmental and sustainability education related to SDG 6. Comparative studies in different cultural contexts, such as those conducted in Taiwan and Sweden, indicate substantial variation in sustainability-related attitudes and behaviors, underscoring the need for context-specific measurement tools ( Berglund et al., 2020).

Further longitudinal and intervention-based studies are also needed to examine whether habits-of-mind–focused educational approaches lead to sustained behavioral and attitudinal change, particularly in rural and under-resourced regions of Indonesia. In addition, deeper investigation into the relationship between cognitive dispositions and sustainability education remains essential. Research on Education for Sustainable Development (ESD) suggests that linking cognitive development with environmental awareness can support pro-sustainability behaviors ( Ampuero et al., 2015; Sood et al., 2022). Expanding international research collaboration may therefore help refine and scale educational frameworks that are both globally informed and locally grounded, contributing to more equitable and context-responsive educational reforms in Indonesia.

## Conclusion

### Summary of findings

This study of research papers reveals that habits of mind are increasingly important to 21st-century skill development. Habits of mind refer to mental, emotional and physical features that help someone become reflective, creative, self-controlled and able to solve problems well ( Ariyati et al., 2020; Oehlman, 2023; Susilowati et al., 2018). It is clear from the analysis that much of the research comes from developed countries, more is being done in Indonesia and both STEM and problem-based learning frameworks use habits of mind widely. Although there has been important progress, not enough research has been done to understand habits of mind in different cultural and economic settings, especially in Indonesia. The overlap of habits of mind and sustainability education creates an interesting direction for further study.

### Implications for education policy

Systematic inclusion of certain habits of mind in both the curriculum and teachers’ professional development is necessary to strengthen and increase the level of innovation among Indonesian workers. Looking at these mental skills early will make it simpler for students to face knowledge challenges and support the achievement of global development goals, including SDG 6. When learners are taught to think critically, they are able to find sustainable answers for water and sanitation, needed to reach Sustainable Development Goal 6 ( Al-Noaimi, 2020; Ortigara et al., 2018).

### Future research directions

Further studies are needed to create culturally appropriate tools for assessment, to study habits of mind methods over time and to review how they influence various groups of learners. Also, more research on linkages between ways of thinking and sustainability education, related to SDG 6, is necessary to drive changes in education theory and practice.

## Ethical approval and consent

Ethical approval and consent were not required.

## Data Availability

All data underlying the results of this study are available in Zenodo https://doi.org/10.5281/zenodo.17176051 ( Nada et al., 2025). Data are available under the terms of the Creative Commons Attribution 4.0 International license (CC-BY 4.0). The repository includes the values used to construct all figures and tables, bibliometric indicator values, extracted records, and accompanying documentation for replication. Supplementary materials, including search strategies, raw bibliometric datasets, and VOSviewer mapping files, are available in Zenodo https://doi.org/10.5281/zenodo.17149790 ( Nada et al., 2025). Data are available under the terms of the Creative Commons Attribution 4.0 International license (CC-BY 4.0).

## References

[ref1] Akçay B : Problem-based learning in science education. *J. Turk. Sci. Educ.* 2009;6(1):26–36. Reference Source

[ref2] Allen W Hyde M Whannel R : Teacher reform in Indonesia: can offshore programs create lasting pedagogical shift? *Asia Pac. J. Teach. Educ.* 2018;46(1):22–37. 10.1080/1359866X.2017.1355051

[ref3] Al-Noaimi MA : Sdg goal 6 monitoring in the Kingdom of Bahrain. *Desalin. Water Treat.* 2020;176:406–427. 10.5004/dwt.2020.25552

[ref4] Alper A : Critical thinking disposition of pre-service teachers. *Egitim ve Bilim.* 2010;35(158):14–27. Reference Source

[ref5] Ampuero DA Miranda C Goyen S : Positive psychology in education for sustainable development at a primary-education institution. *Local Environ.* 2015;20(7):745–763. 10.1080/13549839.2013.869199

[ref6] Ardoin NM Bowers AW Gaillard E : Environmental education outcomes for conservation: A systematic review. *Biol. Conserv.* 2020;241:108224. 10.1016/j.biocon.2019.108224

[ref7] Ariyati E Fitriyah FK : AN INVESTIGATION INTO HABITS OF MIND PROSPECTIVE TEACHER: DO THEY HAVE IT?; [UMA INVESTIGAÇÃO SOBRE OS HÁBITOS DA MENTE PROFESSOR EM PROSPECTIVA: ELES TÊM ISSO?]; [UNA INVESTIGACIÓN SOBRE HÁBITOS DEL PROFESOR MENTE PROSPECTIVO: ¿LO TIENEN?]. *Revista de Gestao Social e Ambiental.* 2024;18(5). 10.24857/rgsa.v18n5-087

[ref8] Ariyati E Susilo H Suwono H : Habits of mind potency of students of prospective biology teacher. *J. Phys. Conf. Ser.* 2020;1567(2):022048. 10.1088/1742-6596/1567/2/022048

[ref10] Ariyati E Susilo H Suwono H : Building students’ habits of mind through process oriented guided inquiry learning. *J. Phys. Conf. Ser.* 2021;1918(5):052077. 10.1088/1742-6596/1918/5/052077

[ref11] Berglund T Gericke N Boeve-de Pauw J : A cross-cultural comparative study of sustainability consciousness between students in Taiwan and Sweden. *Environ. Dev. Sustain.* 2020;22(7):6287–6313. 10.1007/s10668-019-00478-2

[ref12] Bhuyan A Sanguri K Sharma H : Improving the Keyword Co-occurrence Analysis: An Integrated Semantic Similarity Approach. *2021 IEEE International Conference on Industrial Engineering and Engineering Management, IEEM 2021.* 2021;482–487. 10.1109/IEEM50564.2021.9673030

[ref13] Boyle A Wilson E Dimmock K : Space for sustainability?: Sustainable education in the tourism curriculum space. *The Routledge Handbook of Tourism and Hospitality Education.* 2014; pp.519–531. 10.4324/9780203763308-53

[ref14] Carroll D : Examining the Development of Dispositions for Ambitious Teaching: One Teacher Candidate’s Journey. *New Educ.* 2012;8(1):38–64. 10.1080/1547688X.2011.619950

[ref15] Cech F : Exploring emerging topics in social informatics: An online real-time tool for keyword co-occurrence analysis. *Lecture Notes in Computer Science (Including Subseries Lecture Notes in Artificial Intelligence and Lecture Notes in Bioinformatics), 10540 LNCS.* 2017; pp.527–536. 10.1007/978-3-319-67256-4_42

[ref16] Chen RH : Fostering students’ workplace communicative competence and collaborative mindset through an inquiry-based learning design. *Educ. Sci.* 2021;11(1):1–13. 10.3390/educsci11010017

[ref17] Choi S-Y : Industry Insights on Future Convergence Education: A Survey of Key Competencies and Educational Directions. *IEEE Global Engineering Education Conference, EDUCON.* 2024. 10.1109/EDUCON60312.2024.10578909

[ref18] Crick RD : Assessment in Schools – Dispositions. *International Encyclopedia of Education.* Third Edition.2009. 10.1016/B978-0-08-044894-7.01208-2

[ref19] Cusanelli LN Trevallion D : Using technology for productive, creative purpose. *International Journal of Innovation, Creativity and Change.* 2020;13(1):1–12. Reference Source

[ref20] Danús L Muntaner C Krauss A : Differences in collaboration structures and impact among prominent researchers in Europe and North America. *EPJ Data Sci.* 2023;12(1). 10.1140/epjds/s13688-023-00378-6

[ref21] Dolfing R Prins GT Bulte AMW : Strategies to support teachers’ professional development regarding sense-making in context-based science curricula. *Sci. Educ.* 2021;105(1):127–165. 10.1002/sce.21603

[ref22] Dooly Z Duane A O’Driscoll A : Creating and Managing EU Funded Research Networks: An Exploratory Case. *Electron. J. Bus. Res. Methods.* 2022;20(1):pp1–p20. 10.34190/ejbrm.20.1.2556

[ref23] Dwirahayu G Kustiawati D Bidari I : Corresponding Habits of Mind and Mathematical Ability. *J. Phys. Conf. Ser.* 2017;895(1):012013. 10.1088/1742-6596/895/1/012013

[ref24] Dwyer CP Hogan MJ Stewart I : An integrated critical thinking framework for the 21st century. *Think. Skills Creat.* 2014;12:43–52. 10.1016/j.tsc.2013.12.004

[ref25] Erjavec E : Reinforcement of Social Sustainability through Education and Public Intangible Capital. *Challenges on the Path Towards Sustainability in Europe: Social Responsibility and Circular Economy Perspectives.* 2020; pp.253–270. 10.1108/978-1-80043-972-620201014

[ref26] Firdaussy UF Ningsih S Asrawijaya E : Basic education for indigenous peoples in Indonesia: Limiting children’s cultural alienation and loss of identity. *Issues Educ. Res.* 2024;34(3):995–1015. Reference Source

[ref27] Fry CV Lynham J Tran S : Ranking researchers: Evidence from Indonesia. *Res. Policy.* 2023;52(5):104753. 10.1016/j.respol.2023.104753

[ref28] Gloria RY Sudarmin S Indriyanti DR : The effectiveness of formative assessment with understanding by design (UbD) stages in forming habits of mind in prospective teachers. *J. Phys. Conf. Ser.* 2018;983(1):012158. 10.1088/1742-6596/983/1/012158

[ref29] Gray S Sterling EJ Aminpour P : Assessing (social-ecological) systems thinking by evaluating cognitive maps. *Sustainability (Switzerland).* 2019;11(20). 10.3390/su11205753

[ref30] Hallinger P Kovačević J : Applying bibliometric review methods in education: rationale, definitions, analytical techniques, and illustrations. *International Encyclopedia of Education.* Fourth Edition.2022. 10.1016/B978-0-12-818630-5.05070-3

[ref31] Hanushek EA : Economic growth in developing countries: The role of human capital. *Econ. Educ. Rev.* 2013;37:204–212. 10.1016/j.econedurev.2013.04.005

[ref32] Herawan T Anggraini FD Ihalauw JJOI : Visualizing Trends in Tourism Entertainment Researches: Bibliometric Analysis Using the Scopus Database. *Lecture Notes in Networks and Systems, 837 LNNS.* 2024a; pp.498–504. 10.1007/978-3-031-48465-0_67

[ref33] Herawan T Ermawati KC Ihalauw JJOI : Information Visualization of Research Evolution on Innovation in Local Wisdom: A Decade Bibliometric Analysis Using the Scopus Database. *Lecture Notes in Networks and Systems, 837 LNNS.* 2024b; pp.485–491. 10.1007/978-3-031-48465-0_65

[ref34] Hidayati N Idris T : Students’ habits of mind profiles of biology education department at public and private universities in Pekanbaru, Indonesia. *Int. J. Instr.* 2020;13(2):407–418. 10.29333/iji.2020.13228a

[ref35] Hosseini E Alipour-Tehrani M Salemi N : Evolution, Growth, and Maturity of the Thematic Network in the field of Citation Bias. *Scientometrics Research Journal.* 2025;11(1):75–108. 10.22070/rsci.2024.18862.1720

[ref36] Ismail LS : Innovative synergies collaborating with industry, research organizations, and global institutions in higher education. *Artificial Intelligence, Digital Learning, and Leadership: Redefining Higher Education.* 2024;203–217. 10.4018/979-8-3373-0025-2.ch008

[ref37] Janzen S : Advancing the Advanced Search: Improving the Web of Science User Experience with a Spreadsheet Search Query Tool. *Communications in Computer and Information Science, 1580 CCIS.* 2022; pp.292–299. 10.1007/978-3-031-06417-3_40

[ref38] Jayasree V Baby MD : Scientometrics: Tools, Techniques and Software for Analysis. *Indian Journal of Information Sources and Services.* 2019;9(2):116–121. 10.51983/ijiss.2019.9.2.611

[ref39] John M : The Routledge handbook of global sustainability education and thinking for the 21st century. *The Routledge Handbook of Global Sustainability Education and Thinking for the 21st Century.* 2025. 10.4324/9781003171577

[ref40] Karaarslan-Semiz G : Outdoor Education for Sustainability with Systems Thinking Perspective. *Sustainable Development Goals Series*, *Part F2743.* 2022; pp.39–53. 10.1007/978-3-031-09112-4_4

[ref41] Kareem HH Dehham SH Al-Wahid MA : The impact of teaching the creative writing by FOCUS strategy to develop. *Indian J. Public Health Res. Dev.* 2019;10(6):876–880. 10.5958/0976-5506.2019.01390.1

[ref42] Khasseh AA Soheili F Mousavi Chelak A : An author co-citation analysis of 37 years of iMetrics. *Electron. Libr.* 2018;36(2):319–337. 10.1108/EL-09-2016-0191

[ref43] Kusumawardani CA Rosyidi CN Jauhari WA : The evaluation of criteria and subcriteria of research project selection using fuzzy analytical hierarchy process method. *2016 2nd International Conference of Industrial, Mechanical, Electrical, and Chemical Engineering, ICIMECE 2016.* 2017; pp.112–117. 10.1109/ICIMECE.2016.7910429

[ref44] Lander L : Sustainability education: Is thinking the key? *Sustainability (United States).* 2015;8(1):27–31. 10.1089/SUS.2015.0005

[ref45] Levy MA Lubell MN McRoberts N : The structure of mental models of sustainable agriculture. *Nat. Sustain.* 2018;1(8):413–420. 10.1038/s41893-018-0116-y

[ref46] Lim WM Kumar S Donthu N : How to combine and clean bibliometric data and use bibliometric tools synergistically: Guidelines using metaverse research. *J. Bus. Res.* 2024;182:114760. 10.1016/j.jbusres.2024.114760

[ref47] Lin T McNab P : Adaptive support for inductive reasoning ability. *Web-Based Intelligent E-Learning Systems: Technologies and Applications.* 2005a; pp.1–23. 10.4018/978-1-59140-729-4.ch001

[ref48] Lin T McNab P : Supporting inductive reasoning in adaptive virtual learning environment. *Proceedings of the IASTED International Conference on Web-Based Education, WBE 2005.* 2005b; pp.562–566. Reference Source

[ref49] Lower-Hoppe LM Brgoch S Chen Y-J : Inquiry-based learning in action: Theory and practice in higher education. *Handbook of Research on Innovations in Non-Traditional Educational Practices.* 2020; pp.34–59. 10.4018/978-1-7998-4360-3.ch003

[ref50] Makhanya MS : Research in transforming contexts: Ensuring relevance and impact. *International Perspectives on Undergraduate Research: Policy and Practice.* 2020; pp.177–203. 10.1007/978-3-030-53559-9_10

[ref51] Marvi R Foroudi MM : Bibliometric analysis: Main procedure and guidelines. *Researching and Analysing Business: Research Methods in Practice.* 2023; pp.43–54. 10.4324/9781003107774-4

[ref52] Mathews SR Lowe K : Classroom environments that foster a disposition for critical thinking. *Learn. Environ. Res.* 2011;14(1):59–73. 10.1007/s10984-011-9082-2

[ref53] Merigó JM Yang J-B : Accounting Research: A Bibliometric Analysis. *Aust. Account. Rev.* 2017;27(1):71–100. 10.1111/auar.12109

[ref54] Moscardo G : The Importance of Education for Sustainability in Tourism. *CSR, Sustainability, Ethics and Governance.* 2015; pp.1–21. 10.1007/978-3-662-47470-9_1

[ref55] Nada EI Sajidan S Ariani SRD : Bibliometric Dataset for Habits of Mind and SDG 6 in Educational Research. *Zenodo.* 2025. 10.5281/zenodo.17176051 PMC13033131 41913753

[ref56] Nadiroh N Zulfa V Yuliani S : Learning transformation of the 21stcentury curriculum for prospective teacher in term of eco-literacy. *IOP Conf. Ser.: Earth Environ. Sci.* 2021;802(1):012009. 10.1088/1755-1315/802/1/012009

[ref57] Nageye AY Jimale AD Abdullahi MO : Emerging Trends in Data Science and Big Data Analytics: A Bibliometric Analysis. *SSRG Int. J. Electron. Commun. Eng.* 2024;11(5):84–98. 10.14445/23488549/IJECE-V11I5P109

[ref58] Nahadi SA Windani DK : Investigating formative assessment strategy to chemistry habits of mind (CHOM) of buffer solution concept in learning chemistry. *Turk. Online J. Educ. Technol.* 2017;2017(November Special Issue INTE):276–285. Reference Source

[ref59] Nandiyanto ABD Ragadhita R Al Husaeni DN : Research trend on the use of mercury in gold mining: Literature review and bibliometric analysis. *Moroc. J. Chem.* 2023;11(1):1–19. 10.48317/IMIST.PRSM/morjchem-v11i1.36576

[ref60] Narayanan M : Assessment of problem-based learning. *ASEE Annual Conference and Exposition, Conference Proceedings.* 2010. Reference Source

[ref61] Obuseh M Duffy VG : Surgical Human-Robot Interaction: A Bibliometric Review. *Lecture Notes in Computer Science (Including Subseries Lecture Notes in Artificial Intelligence and Lecture Notes in Bioinformatics)*, *13519 LNCS.* 2022; pp.293–312. 10.1007/978-3-031-17618-0_22

[ref62] O’Dwyer A : Blue gold: Clean water and sanitation (SDG 6). *Teaching the Sustainable Development Goals to Young Citizens (10-16 Years): A Focus on Teaching Hope, Respect, Empathy and Advocacy in Schools.* 2024; pp.194–214. 10.4324/9781003232001-12

[ref63] Oehlman N : Using the Habits of Mind as a Reflective Tool. *Fostering Habits of Mind in Today’s Students: A New Approach to Developmental Education.* 2023; pp.213–220. 10.4324/9781003444862-28

[ref64] Olaniyan DA Okemakinde T : Human capital theory: Implications for educational development. *Eur. J. Sci. Res.* 2008;24(2):157–162. Reference Source

[ref65] Ortigara ARC Kay M Uhlenbrook S : A review of the SDG 6 synthesis report 2018 from an education, training, and research perspective. *Water (Switzerland).* 2018;10(10). 10.3390/w10101353

[ref66] Petera P : Strategic management accounting: Bibliometric analysis and ideas for future research. *Proceedings of the 30th International Business Information Management Association Conference, IBIMA 2017 - Vision 2020: Sustainable Economic Development, Innovation Management, and Global Growth, 2017-January.* 2017; pp.4229–4240. Reference Source

[ref67] Portera M : Awareness as a challenge: Learning through our bodies on a planet in crisis. *The Palgrave Handbook of Embodiment and Learning.* 2022; pp.61–73. 10.1007/978-3-030-93001-1_4

[ref68] Portera M : Habits. *Handbook of the Anthropocene: Humans between Heritage and Future.* 2023; pp.1127–1130. 10.1007/9783031259104_184

[ref69] Purcell WM Henriksen H Spengler JD : Universities as the engine of transformational sustainability toward delivering the sustainable development goals: “Living labs” for sustainability. *Int. J. Sustain. High. Educ.* 2019;20(8):1343–1357. 10.1108/IJSHE-02-2019-0103

[ref70] Qablan A Nowfal N Al-Faiadh T : Mapping the representation of four SDGs in international elementary science curriculum and textbooks. *Eurasia J. Math. Sci. Technol. Educ.* 2025;21(3):em2596. 10.29333/ejmste/16042

[ref71] Restrepo-Arango C Urbizagástegui-Alvarado R : Co-words network in Mexican bibliometrics; [Red de co-palabras en la bibliometría Mexicana]. *Investigacion Bibliotecologica.* 2017;31(73):17–45. 10.22201/iibi.24488321xe.2017.73.57845

[ref72] Saa S : MERDEKA CURRICULUM: ADAPTATION OF INDONESIAN EDUCATION POLICY IN THE DIGITAL ERA AND GLOBAL CHALLENGES; [CURRÍCULO MERDEKA: ADAPTANDO A POLÍTICA EDUCACIONAL DA INDONÉSIA À ERA DIGITAL E AOS DESAFIOS GLOBAIS]; [CURRÍCULO MERDEKA: ADAPTANDO A POLÍTICA EDUCACIONAL DE INDONESIA A LA ERA DIGITAL Y A LOS DESAFÍOS GLOBALES]. *Revista de Gestao Social e Ambiental.* 2024;18(3). 10.24857/rgsa.v18n3-168

[ref73] Sáez VM Salomón YP Domínguez Díaz JM : Bibliometric analysis of Spanish research on Communication for Development and Social Change (2014-2020): emerging thematics or field in implosion?; [Análisis bibliométrico de la investigación española sobre Comunicación para el Desarrollo y el Cambio Social (2014-2020): ¿temáticas emergentes o campo en implosión?]. *Transinformacao.* 2023;35. 10.1590/2318-0889202335e226852

[ref74] Sahni S Kaurav RPS : WHAT? WHY? WHEN? HOW? WHERE? OF TECHNOLOGY-BASED BIBLIOMETRIC REVIEW. *Review of Management Literature.* 2023;2:79–101. 10.1108/S2754-586520230000002005

[ref75] Sanneh ES : Systems thinking for sustainable development: Climate change and the environment. *Systems Thinking for Sustainable Development: Climate Change and the Environment.* 2018. 10.1007/978-3-319-70585-9

[ref76] Scherbakova NG Bredikhin SV : Co-authorship network structure analysis. *J. Phys. Conf. Ser.* 2021;2099(1):012055. 10.1088/1742-6596/2099/1/012055

[ref77] Sillet A : Defi nition and use of bibliometrics in research. *Soins.* 2013;58(781):29–30. 10.1016/j.soin.2013.10.002 24558685

[ref78] Singha R Singha S : Application of experiential, inquiry-based, problem-based, and project-based learning in sustainable education. *Teaching and Learning for a Sustainable Future: Innovative Strategies and Best Practices.* 2024; pp.109–128. 10.4018/978-1-6684-9859-0.ch006

[ref79] Sood A Reed H Stanton A : Playponics in India—Local Hydroponics Playground Gardens Utilising Kinaesthetic Learning to Promote Global Sustainable Practices. *Springer Series in Design and Innovation.* 2022;17:435–443. 10.1007/978-3-030-86596-2_31

[ref80] Sprow AH : Higher Education Institutions’ Role in Developing Future Leaders for Sustainability: Incorporating the Inner Development Goals. *Int. J. Soc. Sustain. Econ. Soc. Cult. Context.* 2025;20(1):117–132. 10.18848/2325-1115/CGP/v20i01/117-132

[ref81] Sukoco BM Putra RA Muqaffi HN : Comparative Study of ASEAN Research Productivity. *SAGE Open.* 2023;13(1). 10.1177/21582440221145157

[ref82] Sundar S Gurupandi M : Exploring Strategic Entrepreneurship Research: A Comprehensive Decadal Bibliometric Analysis. *Int. Rev. Manag. Mark.* 2025;15(1):179–192. 10.32479/irmm.17438

[ref83] Susilowati E Hartini S Mayasari T : Profile Habits of Mind Students in Physics Learning. *J. Phys. Conf. Ser.* 2018;1120(1):012055. 10.1088/1742-6596/1120/1/012055

[ref84] Susilowati E Mayasari T Winarno N : Scaffolding learning model to improve habits of mind students. *J. Phys. Conf. Ser.* 2019;1280(5):052064. 10.1088/1742-6596/1280/5/052064

[ref85] Thomas MB Muscat A Zuccolo A : Navigating Pedagogical Innovation in Higher Education: Education Academics’Experiences with Active and Inquiry-Based Learning in Intensive Teaching. *Innov. High. Educ.* 2025. 10.1007/s10755-025-09807-y

[ref86] Valérie D Pierre AG : Bibliometric idicators: Quality masurements of sientific publication. *Radiology.* 2010;255(2):342–351. 10.1148/radiol.09090626 20413749

[ref87] Vira R : Environmental education of young managers. *International Multidisciplinary Scientific GeoConference Surveying Geology and Mining Ecology Management, SGEM.* 2019;19(5.4):139–146. 10.5593/sgem2019/5.4/S22.019

[ref88] Visser TC Coenders FGM Terlouw C : Essential Characteristics for a Professional Development Program for Promoting the Implementation of a Multidisciplinary Science Module. *J. Sci. Teach. Educ.* 2010;21(6):623–642. 10.1007/s10972-010-9212-1

[ref89] Voak A Fairman B Helmy A : Kampus Merdeka: Providing Meaningful Engagement in a Disruptive World. *J. High. Educ. Theory Pract.* 2023;23(8):223–234. 10.33423/jhetp.v23i8.6076

[ref90] Weaver M Fonseca AP Tan H : Systems thinking for sustainability: shifting to a higher level of systems consciousness. *J. Oper. Res. Soc.* 2025;1–14. 10.1080/01605682.2025.2486698

[ref91] Wulandari R Iswara AP Qadafi M : Water pollution and sanitation in Indonesia: a review on water quality, health and environmental impacts, management, and future challenges. *Environ. Sci. Pollut. Res.* 2024;31(58):65967–65992. 10.1007/s11356-024-35567-x 39623134

[ref92] Zerai M Ajailia N : ENGINEERING EDUCATION IN THE ERA OF GLOBAL RESPONSIBILITY. *Proc. Int. CDIO Conf.* 2024;495–505. Reference Source

[ref93] Zreik M : The paradox of educational inequality in Indonesia: Socioeconomic implications and paths towards inclusion. *Socio-Economic Implications of Global Educational Inequalities.* 2023; pp.69–85. 10.4018/979-8-3693-0693-2.ch004

