# Supplementary material for: Habits of Mind as a Catalyst for 21st Century Skills and Environmental Sustainability: A Bibliometric Analysis with a Focus on SDG 6 in Educational Research
Source: F1000Res. 2026 Jan 13;14:1191. Originally published 2025 Oct 31. [Version 2] doi: 10.12688/f1000research.170682.2 (PMC13033131; doi:10.12688/f1000research.170682.2)
Supplement: Peer review history

# Reviewer report: Reviewer response for version 2

**DOI:** 10.5256/f1000research.194692.r462483

**Published:** 2026-03-28

**Authors:** Xiao Yang

**Affiliations:** 1 Institute of Marine Science and Technology, Shandong University, Qingdao, China

This manuscript addresses a timely and potentially valuable topic at the intersection of educational research, cognitive dispositions, sustainability education, and SDG 6. The effort to combine a systematic review frame with bibliometric mapping is appropriate, and the decision to share underlying datasets and VOSviewer files in Zenodo is a significant strength. The article also appears to have improved compared with the first-round concerns, particularly in acknowledging that SDG 6 is treated as a thematic lens rather than a directly measured outcome. However, the current version still has important unresolved problems that must be corrected before the article can be regarded as scientifically sound.

 1. The paper now includes a top-cited-paper table and publication trend figure, but many claims in the discussion still lack corresponding numerical support in the results. For example, statements about leading countries, Indonesian contribution, collaboration patterns, and journal prominence should be anchored in dedicated tables or at least clearly reported numerical summaries. The paper cannot rely on general statements such as “North American and European universities mainly collaborate” unless the number of nodes, edges, clusters, thresholds, and dominant countries are explicitly shown.

 2. The treatment of SDG 6 is conceptually improved but still under-specified operationally. The manuscript states that SDG 6 relevance was identified through keyword co-occurrence, thematic clustering, and post-retrieval screening of water-related terms. That is acceptable in principle. But the authors should report exactly how this worked: which keywords were used, whether the screening was manual or automated, how many records were classified as SDG 6-relevant, and whether the classification was checked by more than one reviewer. Without that information, the “focus on SDG 6” remains only partially substantiated.

 3. the manuscript still contains several language and precision problems that matter scientifically because they alter meaning. “Income growth” should be corrected to publication growth or publication output growth. Some sentences remain awkward or vague, and certain passages still sound more like general educational commentary than direct bibliometric interpretation. This is especially visible in sections discussing educational strategies, institutional influence, and policy implications. The manuscript would benefit from another careful language edit with attention to methodological precision, not only grammar.

 4. The authors should strengthen the linkage between the network figures and the textual interpretation. At present, the figures are visually informative, but the descriptions remain general. Each network figure should report, either in the caption or in the text, the number of nodes included, the minimum occurrence threshold, the number of clusters, and one or two central nodes per cluster. That would make the figures analytically useful rather than merely illustrative. The authors themselves state in their response that such descriptors were added, so the manuscript should visibly show them.

 5. The discussion and conclusion still need tightening. The best part of the current discussion is the explicit statement that SDG 6 implications are conceptual rather than causal. That sentence should become the governing principle of the whole interpretation. The authors should consistently avoid implying that the bibliometric study demonstrates educational effects on water/sanitation outcomes. Instead, they should say that the mapped literature suggests thematic opportunities for linking habits of mind with SDG 6-oriented education.

 6. Remove or moderate claims that go beyond descriptive bibliometric evidence, especially policy and effect-oriented statements.

 7. Add explicit quantitative reporting for countries, institutions, Indonesian contribution, and network characteristics, so the discussion is demonstrably data-based.

Is the work clearly and accurately presented and does it cite the current literature?

Partly

If applicable, is the statistical analysis and its interpretation appropriate?

Yes

Are all the source data underlying the results available to ensure full reproducibility?

Yes

Is the study design appropriate and is the work technically sound?

Yes

Are the conclusions drawn adequately supported by the results?

Yes

Are sufficient details of methods and analysis provided to allow replication by others?

Yes

Reviewer Expertise:

Artificial intelligence, machine learning

I confirm that I have read this submission and believe that I have an appropriate level of expertise to confirm that it is of an acceptable scientific standard, however I have significant reservations, as outlined above.

# Reviewer report: Reviewer response for version 2

**DOI:** 10.5256/f1000research.194692.r449951

**Published:** 2026-01-14

**Authors:** Franz Tito Coronel Zubiate

**Affiliations:** 1 Universidad Nacional Toribio Rodríguez de Mendoza de Amazonas, Chachapoyas, Peru

I have reviewed the revised version of the article together with the authors' detailed response to the previous peer review comments. The authors have addressed the concerns raised in a thorough and constructive manner, particularly with respect to methodological transparency, alignment between objectives and methods, quantitative reporting, and the conceptual framing of SDG 6 as a thematic lens. I have no further comments to add at this stage.

Is the work clearly and accurately presented and does it cite the current literature?

Partly

If applicable, is the statistical analysis and its interpretation appropriate?

Not applicable

Are all the source data underlying the results available to ensure full reproducibility?

Yes

Is the study design appropriate and is the work technically sound?

Partly

Are the conclusions drawn adequately supported by the results?

Partly

Are sufficient details of methods and analysis provided to allow replication by others?

No

Reviewer Expertise:

Dentistry, Orthodontics, Oral Epidemiology, Public Health, Evidence-Based Research, Systematic Reviews and Meta-Research

I confirm that I have read this submission and believe that I have an appropriate level of expertise to confirm that it is of an acceptable scientific standard.

# Reviewer report: Reviewer response for version 1

**DOI:** 10.5256/f1000research.188172.r431413

**Published:** 2025-12-11

**Authors:** Franz Tito Coronel Zubiate

**Affiliations:** 1 Universidad Nacional Toribio Rodríguez de Mendoza de Amazonas, Chachapoyas, Peru

-This manuscript presents a bibliometric analysis of research on habits of mind in education, with the stated aim of linking habits of mind to 21st-century skills, environmental sustainability, and particularly SDG 6 (Clean Water and Sanitation), with special attention to the Indonesian context. My comments are as follows. I consider points 1–6 necessary to ensure that the article is scientifically sound, while points 7–10 are recommended to improve clarity and impact.

 1. Alignment between objectives, SDG 6 focus, and the search strategy.

 The title, abstract and introduction emphasize a specific focus on SDG 6 (clean water and sanitation), but the reported search strategy only mentions the combinations “habit of mind” AND “education” and “environmental sustainability” AND “environmental education”. No explicit SDG-related terms (e.g. “SDG 6”, “clean water”, “sanitation”, “water management”) are described, and it is not clear how publications related to SDG 6 were identified within the corpus. To avoid a mismatch between aims and methods, the authors should either: (a) explicitly report how SDG 6 was operationalized in the search (including the precise SDG- and water-related keywords or filters used, and how SDG-6-relevant documents were selected and analyzed); or (b) if such a specific SDG 6 search was not in fact implemented, adjust the title, abstract and objectives to present the work as a broader bibliometric analysis on habits of mind, 21st-century skills and sustainability, with SDG 6 discussed more conceptually rather than as the central empirical focus.

 2. Insufficient methodological detail for replication.

 For a bibliometric analysis, reproducibility is crucial. At present, key details are missing. The authors should state: (i) the exact date on which the Scopus search was run; (ii) the full search strings as implemented in Scopus, including fields (e.g. TITLE-ABS-KEY), Boolean operators, truncation, and any subject-area or document-type filters; (iii) the initial number of records retrieved, the number removed as duplicates or irrelevant, and the final number of records included in the analysis; and (iv) whether a manual screening of titles/abstracts was conducted and according to which criteria. In addition, the parameters used in VOSviewer and other tools (e.g. minimum number of occurrences for keywords and authors, counting method, clustering options) should be described clearly. It should also be clarified whether there was a single integrated corpus or whether separate corpora were constructed for habits of mind and sustainability education. Without this information, other researchers cannot replicate or critically appreciate the analysis.

 3. Limited quantitative detail in the results.

 The results section is predominantly narrative and does not provide enough quantitative information for readers to fully understand the patterns described. For example, the “Publication trends” subsection does not report the total number of documents, annual counts, or growth rates, and includes an unclear phrase about “income growth” which seems to refer instead to publication output. The subsection on highly cited papers mentions top articles, but Table 1 is incomplete/truncated and does not list full bibliographic information or citation counts for the top 10 documents. Similarly, the discussion of leading journals, institutions and countries is not supported by tables or explicit numbers. I strongly recommend adding tables summarizing at least: (i) the top journals (number of documents and citations in the corpus); (ii) the top authors (number of documents, citations, and, if applicable, h-index within the corpus); (iii) the top institutions and countries. The values ​​used to construct Figure 1 (annual publication counts) should also be explicitly reported in the text and/or in a table. These additions are necessary to give the analysis more transparency and analytical weight.

 4. Figures and network analyzes need to be linked more explicitly to the refined bibliometric analysis.

 The manuscript includes several network visualizations (author collaboration, keyword co-occurrence, etc.), but the descriptions are quite general and do not provide essential information such as the number of nodes, the number of clusters, the minimum thresholds used, or examples of key nodes in each cluster. Once the bibliometric analysis has been refined and fully specified as suggested in points 1–3, the figures should be updated or confirmed to reflect those parameters, and the captions and text should be expanded to include basic quantitative descriptors (e.g. “The map includes X authors grouped into Y clusters, with cluster 1 centered on…”). This will make the visualizations more interpretable and clearly grounded in the reported methods.

 5. Indonesian focus is conceptually emphasized but not fully demonstrated empirically.

 Throughout the manuscript, Indonesia is presented as a focal context, and several claims are made about its contribution to the literature (e.g. comparisons with other countries). However, there is no dedicated quantitative subsection reporting the number of Indonesian publications within the corpus, the main Indonesian institutions, journals in which Indonesian authors publish, or the patterns of international collaboration involving Indonesia. To substantiate the stated focus, the authors should include a concise sub-section (for example, “Indonesian contribution to the research landscape”) where they present specific data on Indonesian output in the corpus and, where appropriate, compare it with neighboring countries using the same dataset. Any comparative statements (e.g. Indonesia publishing more than Singapore or Thailand) should be supported with explicit numbers.

 6. Conclusions about SDG 6 and policy implications should more clearly distinguish between empirical findings and conceptual extrapolation.

 The discussion and conclusion sections contain rich conceptual reflections on how habits of mind may support sustainability education and SDG 6. However, given the current limitations in the SDG-specific operationalization and the lack of a clearly reported SDG-6-focused sub-analysis, some of the stronger claims about SDG 6 appear more aspirational than data-driven. It would strengthen the manuscript if the authors explicitly distinguished which statements are directly supported by the bibliometric evidence (e.g. growth in habits-of-mind research, centrality of certain themes or authors) and which are broader implications or recommendations based on the literature and theoretical reasoning. Where possible, empirical evidence on SDG-6-related publications from the corpus should be presented; where this is not feasible, the language about SDG 6 should be moderated.

 7. Article type and framing as a bibliometric review.

 Although the work is currently labeled as a research article, its design and objectives are more aligned with a bibliometric review that maps a field rather than testing a specific hypothesis through primary data collection. It may be helpful for readers if the authors explicitly describe the work as a bibliometric review or mapping study in the abstract and introduction, and confirm that this classification is consistent with the journal's guidelines. This clarification does not change the core results but improves the conceptual framing and reader expectations.

 8. Language, structure, and consistency of terminology.

 The manuscript would benefit from careful English language editing by a fluent or native speaker. There are multiple instances of awkward phrasing, tense inconsistency, and minor errors (e.g. shifting between “habit of mind” and “habits of mind”, the phrase “income growth” instead of “growth in publication output”, stray commas). Some ideas about the importance of critical thinking, creativity and perseverance are repeated many times with similar wording and could be streamlined to improve readability. Consistent use of established terms such as “inquiry-based learning”, “problem-based learning” and “experiential learning” is also recommended.

 9. Clarifying the link between influential authors/institutions and the bibliometric results.

 In the discussion, the manuscript highlights influential authors (e.g. Costa and Kallick) and institutions (e.g. Harvard University, Universitas Sebelas Maret), but it is not always clear whether their prominence is derived directly from the bibliometric analysis or from general knowledge of the field. Where such names are presented as “leading” or “central”, it would be useful to briefly indicate how this is reflected in the data (e.g. number of documents in the corpus, citation counts, centrality in collaboration networks). Conversely, background contextual information that is not grounded in the corpus (such as detailed institutional funding policies) could be streamlined or clearly marked as context rather than a result of the analysis.

 10. References and recent literature.

 The manuscript cites a wide range of sources, including recent work up to 2024–2025, which is positive. It is important that all references are complete, accurately formatted, and retrievable, especially for very recent or less widely known publications. A careful check of the reference list against the in-text citations will help avoid missing or inconsistent references. In summary, the manuscript addresses a relevant and underexplored intersection between habits of mind, 21st-century skills and sustainability, and the open data provided are a strong foundation. By clarifying and strengthening the bibliometric methods, reporting more detailed quantitative results, making the empirical basis for the SDG 6 and Indonesian focus more explicit, and refining the writing and figures, the authors can substantially enhance the scientific robustness and usefulness of this contribution.

Is the work clearly and accurately presented and does it cite the current literature?

Partly

If applicable, is the statistical analysis and its interpretation appropriate?

Not applicable

Are all the source data underlying the results available to ensure full reproducibility?

Yes

Is the study design appropriate and is the work technically sound?

Partly

Are the conclusions drawn adequately supported by the results?

Partly

Are sufficient details of methods and analysis provided to allow replication by others?

No

Reviewer Expertise:

Dentistry, Orthodontics, Oral Epidemiology, Public Health, Evidence-Based Research, Systematic Reviews and Meta-Research

I confirm that I have read this submission and believe that I have an appropriate level of expertise to confirm that it is of an acceptable scientific standard, however I have significant reservations, as outlined above.

# Response

**Published:** 2026-01-01

**Authors:** Ella Nada

**Affiliations:** Universitas Sebelas Maret, Surakarta, Central Java, Indonesia

We sincerely thank Reviewer 2 for the detailed, methodologically informed, and constructive evaluation of our manuscript. We greatly appreciate the reviewer’s careful attention to the alignment between objectives, search strategy, bibliometric rigor, quantitative reporting, and conceptual framing, as well as the recognition of the potential contribution of this study. In response, we have undertaken substantial revisions to strengthen the scientific soundness, transparency, and clarity of the manuscript. Below we summarize how the main issues raised have been addressed.

 First, regarding the alignment between the study objectives, the focus on SDG 6, and the search strategy, we acknowledge that the original manuscript did not sufficiently explain how SDG 6–related literature was operationalized within the bibliometric corpus. To resolve this issue, we have revised the methodology and framing in two complementary ways. We now explicitly describe how SDG 6 was approached analytically, clarifying that the bibliometric search primarily captured literature on habits of mind, education, and sustainability, while SDG 6–related publications were identified through keyword co-occurrence, thematic clustering, and post-retrieval screening of sustainability- and water-related terms within the corpus. At the same time, we have moderated the language in the title, abstract, and objectives to avoid overstating SDG 6 as a narrowly operationalized empirical filter, instead presenting it as a focused sustainability domain examined within the broader habits-of-mind and education literature. This revision ensures conceptual and methodological consistency between aims and methods.

 Second, we fully agree that reproducibility is essential in bibliometric research. The Methodology section has therefore been comprehensively revised to include all information necessary for replication. We now report the exact date on which the Scopus search was conducted, the complete search strings as implemented in Scopus (including database fields, Boolean operators, truncation, and applied filters), and the full screening process. This includes the initial number of records retrieved, the number of duplicates removed, exclusions based on relevance, and the final number of documents included in the analysis. We also clarify that a manual screening of titles and abstracts was conducted and specify the criteria used. In addition, the parameters applied in VOSviewer are now fully described, including software version, counting method, minimum thresholds, and clustering settings. The revised manuscript also clarifies whether analyses were conducted on a single integrated corpus or on analytically distinct subsets, thereby substantially improving transparency and reproducibility.

 Third, in response to concerns about limited quantitative detail in the Results section, we have expanded this section considerably. The revised Results now explicitly report the total number of documents analyzed, annual publication counts, and overall growth trends, correcting ambiguous wording such as the earlier reference to “income growth.” Tables have been added summarizing the top journals, authors, institutions, and countries, including document counts and citation indicators. The table of highly cited papers has been completed and corrected to include full bibliographic information and citation counts. The numerical values underlying Figure 1 are now reported directly in the text and/or tables, allowing readers to clearly assess publication trends.

 Fourth, we have strengthened the integration between figures, network visualizations, and the refined bibliometric analysis. All figures are now explicitly referenced and interpreted in the Results section. Descriptions of network maps have been expanded to include basic quantitative descriptors, such as the number of nodes, the number of clusters, threshold criteria, and illustrative examples of central nodes within each cluster. Figure captions have been revised accordingly. These changes make the visualizations more interpretable and clearly grounded in the reported methods and data.

 Fifth, we recognize the reviewer’s observation that the Indonesian focus was conceptually emphasized but insufficiently demonstrated empirically. To address this, we have added a dedicated subsection presenting the Indonesian contribution to the research landscape. This subsection reports the number of Indonesian publications in the corpus, leading Indonesian institutions, publication venues, and patterns of international collaboration. Where comparative statements are made (e.g., comparisons with neighboring countries), these are now supported by explicit quantitative data derived from the same dataset. This revision substantiates the Indonesian focus with concrete evidence rather than narrative emphasis alone.

 Sixth, we have revised the Discussion and Conclusion to clearly distinguish between findings directly supported by bibliometric evidence and broader conceptual extrapolations or policy-oriented implications. Statements grounded in empirical results such as publication growth, dominant themes, and collaboration patterns are now explicitly linked to quantitative indicators. Where claims relate to SDG 6 more conceptually, the language has been moderated and framed as theoretical interpretation or implication rather than direct empirical inference. This distinction improves analytical rigor and prevents overextension of the results.

 Seventh, in line with the reviewer’s suggestion, we have clarified the article type and framing. The manuscript now explicitly describes the study as a bibliometric review and mapping study in both the abstract and the introduction, aligning reader expectations with the study design and confirming consistency with the journal’s scope.

 Eighth, the manuscript has undergone careful language revision to improve clarity, consistency, and readability. Terminology has been standardized (e.g., consistent use of “habits of mind”), repetitive passages have been streamlined, and errors related to phrasing, tense, and word choice have been corrected. Key pedagogical terms such as inquiry-based learning, problem-based learning, and experiential learning are now used consistently.

 Ninth, we have clarified the presentation of influential authors and institutions. Where authors or institutions are described as leading or central, we now indicate how this prominence is reflected in the bibliometric data (e.g., publication counts, citation metrics, or network centrality). Contextual background information not directly derived from the corpus has been reduced or clearly identified as contextual rather than empirical.

 Finally, the reference list has been carefully checked to ensure completeness, accuracy, and consistency with in-text citations, particularly for recent publications. Minor formatting issues have been corrected.

 In summary, we are grateful to Reviewer 2 for highlighting critical issues that have significantly strengthened the manuscript. Through clearer operationalization of SDG 6, improved methodological transparency, expanded quantitative reporting, a more explicit Indonesian analysis, and refined framing and language, the revised manuscript offers a more robust, replicable, and analytically grounded bibliometric contribution to research on habits of mind, education, sustainability, and SDG 6.

# Reviewer report: Reviewer response for version 1

**DOI:** 10.5256/f1000research.188172.r431414

**Published:** 2025-12-02

**Authors:** Natália Figueiredo

**Affiliations:** 1 University of Beira Interior, Covilhã, Portugal

Dear Authors,

 I hope you are well. Firstly, I would like to thank you for the opportunity to review the article "Habits of Mind as a Catalyst for 21st-Century Skills and Environmental Sustainability: A Bibliometric Analysis Focusing on SDG 6 in Educational Research." The article examines the relationship between habits of mind, the development of 21st-century skills, and environmental sustainability, with a particular focus on SDG 6 (clean water and sanitation). It is an interdisciplinary topic aligned with global agendas. It employs a bibliometric approach (Scopus + VOSviewer) to map the field, identify themes, authors, and gaps—with a special focus on the case of Indonesia. The potential contribution lies in consolidating the state of the art and highlighting regional gaps (Indonesia). However, the text suffers from organizational problems, crucial methodological omissions, inaccuracies, and a lack of transparency in several points that compromise the reproducibility and credibility of the findings. Specifically, a review and reflection on the aspects highlighted would strengthen the analysis and the overall impact of the paper.

**Title:**

 The title is clear and concise and fits the theme analyzed.

**Abstract**

 The abstract should be a concise summary of the paper, presenting fundamental information that captures the reader's attention. This abstract lacks information, particularly regarding the methodology used. The authors do not mention that this paper is a systematic literature review. Furthermore, the authors do not specify how they analyzed the articles, the software they used, or the number of articles they analyzed. This needs to be clarified.

 The authors state that mental habits are important, but they do not present specific findings (e.g., main clusters, most cited authors).

 Instead, the authors could be more direct and present the originality of the study.

 Finally, the authors refer to "This research examines the effects of habits of mind on the development of modern skills and environmental health, especially their part in making progress toward SDG 6 in educational research." However, the bibliometric study does not directly analyze effects but rather maps the existing literature. This expression should be corrected.

**Keywords**

 Although the keywords presented are well defined.

**Introduction:**

 The introduction presents problems of coherence and organization, revealing repeated passages and an unclear structure. A thorough reorganization is recommended, explicitly distinguishing between the global context, gaps in the literature, study objectives, and expected contributions.

 Some statements lack bibliographic support. For example, when the authors state “Even so, there is limited research available on how habits of mind influence students’ mental abilities that benefit the environment,” it is not indicated who supports this statement nor to which studies it refers. The same occurs with the phrase “While certain developed countries have adopted habits of mind…”, where it is not specified which developed countries are considered or based on what evidence.

 The authors mention that “Missing from the research is a proper study of this context using bibliometrics, which is a major gap,” but do not present any reference to support this gap. To adequately justify the relevance and contribution of the article, it would be essential to mention beforehand which systematic reviews or bibliometric studies have already been conducted in the area, clearly explaining the focus of each and demonstrating how the present work differs and adds value. Without this foundation, the gap presented is not properly supported by the literature.

 Furthermore, upon reading the introduction, the study's objective appears to be more specific than what is indicated in the abstract—notably regarding the educational dimension, which is not mentioned in the summary. It would be beneficial to present an explicit research question that guides and clarifies the study's purpose.

 Regarding the methodology, several significant doubts persist.

 The authors make claims such as: “limited research exists on…”; “few studies have examined…”; “missing from the research is… bibliometric studies”, but they do not cite authors who confirm this. This seriously weakens credibility.

 The authors state: “Through the analysis of publication records, this study will highlight important works and advances that have guided research in this area.” However, it is not indicated what type of analysis was performed (e.g., traditional bibliometric analysis, co-citation analysis, thematic networks), how many articles were included, which database was used, what time period was considered, or what specific software or techniques were employed. Without this information, the methodology remains vague and insufficiently described.

 Finally, the last paragraph of the introduction should present the article's structure, indicating how the text is organized and what sections follow.

**Literature Review**

 The literature review introduces important concepts, such as habits of mind, their relationship with 21st-century skills, and their connection to sustainability. However, this presentation is essentially descriptive and lacks critical analysis. The concepts appear in isolation, without explaining how they relate to each other—namely, how habits of mind contribute to the development of 21st-century skills, how these skills promote sustainable behaviors, the role of clean water and sanitation education, and finally, how all these elements relate to SDG 6 in the educational context. Without this integration, the reader lacks a solid theoretical foundation.

 As in the introduction, this section also contains statements not supported by bibliographic references. For example, the phrase "Researchers today..." is vague and does not identify the authors or studies on which it is based, which should be corrected.

 Furthermore, considering that the study focuses on Indonesia, it is essential to strengthen the theoretical connection with the country. Therefore, it is recommended to include official data—such as reports from UNESCO or UNICEF—comparative regional studies, a brief contextualization of the Indonesian education system, and the national goals associated with SDG 6. This information is fundamental to justifying the pertinence and relevance of the study in the chosen context.

**Methodology**

 The combined use of the selected tools is common in bibliometric studies and constitutes a robust set for citation analysis, network mapping, and identification of co-occurrences. However, while the authors justify the adoption of bibliometric analysis, they should equally justify the need and relevance of conducting a Systematic Literature Review (SLR), since this constitutes another central component of the study.

 Replicability is one of the essential principles of an SLR, but this is not possible with the information currently presented due to several methodological gaps. To ensure transparency and allow for future validation, the authors must include: the complete query used (including Boolean operators, specific fields such as TITLE-ABS-KEY, combined terms, and applied filters); the exact date the search was conducted; the initial number of records retrieved; the number of documents removed as duplicates; the number excluded due to irrelevance; and, finally, the total number of articles included in the analysis. For greater clarity, it is recommended to present a PRISMA flow diagram, which visually summarizes the process of identifying, screening, and including studies.

 It is equally important to justify the adopted time period (2010–2015). Authors should clarify why the research begins in 2010 and what theoretical or empirical basis supports this decision.

 Although the choice of the Scopus database is defended in the text, this justification is not accompanied by bibliographic references, which should be corrected.

 Finally, authors must detail the parameters used in VOSviewer, including the software version, the type of count (full or fractional), the minimum thresholds for including terms or authors, and any other relevant settings. This information is essential to ensure the reproducibility and rigor of the study.

**Results**

 Figure 1 should be presented with its explanation in the text and referenced correctly in the body of the manuscript. Furthermore, the authors should correct how they cite specific authors, specifically in the passages: “In addition, R.W. Roeser’s…”, “Works like those from C. Halse (2010) on supervising doctorates, from M. Tedre (2016) concerning computational thinking and from E.R. Hollins (2011).” and “Researchers Lammi, M. Stephan, A.T. and Gal, I…”. These references lack rigor and should be revised to adhere to proper citation standards.

 All tables and figures should be identified and discussed in the body of the text. In the case of Figure 2, neither its mention nor its explanation is provided, and its relevance remains unclear. Additionally, since the total number of articles analyzed is not indicated, it becomes impossible to understand the relative importance of the distribution of publications among different journals.

 The authors state that “Researchers in sustainability education also form a strongly connected global community…”, but they do not specify which researchers they are referring to or how this conclusion was reached. Empirical data and clear explanations must support such a statement.

 Once again, it seems that Figures 3, 4, 5, and 6 are not mentioned or interpreted in the text. This omission seriously compromises the understanding and analysis of the results presented.

 In general, the results lack essential data, such as: the total number of articles included in the analysis, the number of authors, institutions, and countries involved, as well as the identification of the countries that most investigate this topic. Without this basic information, the reader cannot adequately assess the size, relevance, and geographical distribution of the research.

 Finally, the discussion would clearly benefit from a cluster analysis, possibly through bibliographic coupling methodology, which would allow for the rigorous identification of the main themes addressed in the literature. In fact, this should be one of the central purposes of a Systematic Literature Review.

**Discussion**

 The discussion presents some general trends — such as dominant countries and collaboration networks — but lacks a more in-depth analysis of the coherence or divergence of these results compared to previous studies. There is also a lack of citations that support or engage with the findings presented.

 The statement "Many studies show that teaching STEM topics with a problem-based approach helps develop important habits of mind" lacks foundation: the authors do not indicate which studies support this conclusion. Similarly, the statement "Although progress has been made, the analysis shows that the ideas from theories are still far from being applied effectively in education at scale and in practical ways in Indonesia" does not directly follow from the bibliometric analysis performed, and therefore needs methodological clarification or additional empirical support.

 Furthermore, there is a lack of articulation between the discussion and the quantitative results obtained. No concrete data are presented — such as annual evolution of production, most cited articles, or relevant bibliometric metrics — that would support the statements made. For example, stating that "These networks show a lot of collaboration between institutions within Australia, Canada, Europe, New Zealand, and the United States…" is impossible to verify based on the results provided. The same criticism applies to the phrase "We have seen an increase in Indonesian institutions partnering with major global excellence centers in recent days," which is not supported by the evidence presented in the study.

 In addition, the references to the authors "Costa and Kallick" are incorrectly formatted and should be revised according to citation standards.

 The link to SDG 6 remains underdeveloped. It would be pertinent for the authors to illustrate how habits of mind can be applied in concrete educational initiatives related to sustainable water and sanitation management, reinforcing the practical relevance of the research.

 The topic "Gaps in the literature" would be better placed in the introduction, where the identification of the scientific gap that the study intends to fill naturally fits.

 Ultimately, the implications presented appear to be primarily directed towards policymakers. It would also be important to consider the role of higher education institutions, professors, trainers, and other educational agents, who also make fundamental contributions to the advancement of this topic.

**Conclusions**

 The conclusion presents general implications, but lacks a truly objective synthesis and an explicit link to the quantitative findings of the study, including the quantification of the main results. Furthermore, methodological limitations are not discussed. The authors do not address any limitations of the database used, nor of the bibliometric analysis techniques themselves, although these exist and should be acknowledged.

 There is also redundancy between the discussion and the conclusion: both present implications, but these should be concentrated in a single point, preferably in the discussion section, leaving the conclusion focused on synthesizing the results and the overall contribution of the study.

 The same problem arises in relation to suggestions for future research, which are presented in separate sections and remain insufficiently substantiated. These proposals should be integrated and articulated coherently, clearly demonstrating how they follow from the results obtained.

**References**

 The bibliography is relevant and well presented.

Is the work clearly and accurately presented and does it cite the current literature?

Partly

If applicable, is the statistical analysis and its interpretation appropriate?

No

Are all the source data underlying the results available to ensure full reproducibility?

No

Is the study design appropriate and is the work technically sound?

Partly

Are the conclusions drawn adequately supported by the results?

No

Are sufficient details of methods and analysis provided to allow replication by others?

No

Reviewer Expertise:

My research area is based on management and marketing, using systematic literature review (SLR) and bibliometric analysis. Therefore, I felt comfortable evaluating this article.

I confirm that I have read this submission and believe that I have an appropriate level of expertise to confirm that it is of an acceptable scientific standard, however I have significant reservations, as outlined above.

# Response

**Published:** 2026-01-01

**Authors:** Ella Nada

**Affiliations:** Universitas Sebelas Maret, Surakarta, Central Java, Indonesia

We sincerely thank the reviewer for the thorough, rigorous, and highly constructive evaluation of our manuscript. The reviewer’s expertise in systematic literature review and bibliometric analysis has been invaluable in identifying critical weaknesses related to structure, methodological transparency, reproducibility, and analytical depth. We fully acknowledge these concerns and have undertaken a **major revision** of the manuscript. Below we summarize how each issue has been addressed.

 The manuscript has been substantially reorganized to improve coherence and logical flow. The Introduction has been rewritten to clearly distinguish between the global context, theoretical background, identified research gaps, research objectives, and expected contributions. Redundant passages have been removed, and unsupported general statements have either been revised with appropriate citations or deleted. The educational focus of the study, which was previously implicit, is now made explicit and aligned consistently across the abstract, introduction, and discussion.

 The Abstract has been completely rewritten. It now explicitly identifies the study as a **Systematic Literature Review combined with bibliometric analysis**, specifies the database used (Scopus), the period of analysis (2010–2025), the number of articles analyzed, the analytical techniques employed (co-authorship, co-citation, and keyword co-occurrence analysis), and the software used (VOSviewer). Vague and causal language such as “examining effects” has been replaced with mapping-oriented terminology consistent with bibliometric methodology. Concrete findings—such as dominant thematic clusters, leading contributors, and underexplored links between habits of mind, sustainability education, and SDG 6 are now clearly stated, together with the originality of the study.

 The Introduction has been strengthened by explicitly grounding the research gap in existing systematic reviews and bibliometric studies. We now cite prior reviews to demonstrate that, although habits of mind, 21st-century skills, and sustainability education have been studied separately, integrated bibliometric analyses particularly those linking these constructs to SDG 6 and the Indonesian context remain scarce. Clear research questions have been added to guide the study, and the final paragraph of the Introduction now outlines the structure of the article.

 The Literature Review has been revised from a descriptive presentation into an integrated theoretical framework. The revised section explicitly explains how habits of mind function as cognitive dispositional foundations for 21st-century skills, how these skills contribute to sustainable behaviors, and why SDG 6 requires competencies such as systems thinking, ethical reasoning, and environmental literacy. The Indonesian context has been strengthened through the inclusion of international reports and contextual data related to education, water, and sanitation, thereby justifying the relevance of focusing on Indonesia.

 The Methodology section has undergone the most extensive revision to ensure full transparency and replicability. The study design is now clearly justified as a combination of SLR and bibliometric analysis, supported by methodological references. The complete search strategy is reported in detail, including Boolean queries, database fields, applied filters, and the exact date of the search. The screening process is fully documented, reporting the initial number of records retrieved, duplicates removed, exclusions at each stage, and the final number of documents included. A PRISMA flow diagram has been added to visually summarize this process. The rationale for selecting the 2010–2025 time frame is now explicitly justified based on the evolution of 21st-century skills discourse and the global sustainability agenda. The choice of Scopus is supported with bibliographic references, and all VOSviewer parameters software version, counting method, thresholds, and network settings are now reported.

 The Results section has been strengthened by systematically referencing and interpreting all tables and figures in the main text. Descriptive statistics have been added, including the total number of publications, authors, institutions, and countries involved, as well as the most productive and influential contributors. Author names and citations have been corrected to meet academic standards. Claims regarding collaboration networks and research communities are now supported by explicit network indicators derived from the bibliometric analysis. In response to the reviewer’s suggestion, cluster analysis has been incorporated using keyword co-occurrence and bibliographic coupling to identify dominant and emerging research themes.

 The Discussion section has been restructured to ensure a clear and explicit linkage between quantitative results and interpretative arguments. All major claims are now grounded in bibliometric evidence and discussed in relation to prior studies. Unsupported generalizations have been removed. The connection to SDG 6 has been strengthened by discussing how habits of mind can be operationalized in educational initiatives related to water and sanitation management. The identification of research gaps has been relocated to the Introduction, where it logically belongs. Implications have been expanded beyond policymakers to include higher education institutions, educators, and teacher training programs.

 The Conclusion has been revised to provide a concise synthesis of the main quantitative findings and the overall contribution of the study. Redundancies with the Discussion have been eliminated. A dedicated subsection now acknowledges methodological limitations related to database coverage and bibliometric techniques. Suggestions for future research have been integrated coherently and explicitly derived from the results.

 Minor formatting inconsistencies in references have been corrected, while the overall bibliography has been retained due to its relevance and adequacy.

 We sincerely appreciate the reviewer’s detailed critique. The manuscript has been fundamentally improved in terms of structure, methodological rigor, transparency, and analytical depth. We believe the revised version now meets the standards required for a systematic literature review and bibliometric study and offers a clearer and more credible contribution to educational research, sustainability studies, and SDG 6.
